# Safety Irradiation Parameters of Nd:YAP Laser Beam for Endodontic Treatments: An In Vitro Study

**DOI:** 10.1155/2016/4741516

**Published:** 2016-06-13

**Authors:** A. Namour, S. Geerts, T. Zeinoun, R. De Moor, S. Nammour

**Affiliations:** ^1^EMDOLA, Department of Dental Science, University of Liège, Quai G. Kurth, P.O. Box 45, 4020 Liège, Belgium; ^2^Division of Conservative and Adhesive Dentistry, Department of Dentistry, University of Liège, Quai G. Kurth, P.O. Box 45, 4020 Liège, Belgium; ^3^Department of Oral and Maxillofacial Surgery, Faculty of Dentistry, Lebanese University, Hadath Campus, Beirut, Lebanon; ^4^Department of Operative Dentistry and Endodontology, Ghent Dental Laser Center, Dental School, Ghent University, 9000 Ghent, Belgium

## Abstract

*Objective*. Nd:YAP laser has several potentialities of clinical applications in endodontics. The aim of our study is to determine the safety range of irradiation parameters during endodontic application of Nd:YAP laser that can be used without damaging and overheating the periodontal tissue.* Material and Methods*. Twenty-seven caries-free single-rooted extracted human teeth were used. Crowns were sectioned to obtain 11 mm root canal length. Temperature increases at root surfaces were measured by a thermocouple during Nd:YAP laser irradiation of root canals at different energy densities. Canal irradiation was accomplished with a circular and retrograde movement from the apex until the cervical part of the canal during 10 seconds with an axial speed of 1 mm/s. Each irradiation was done in a canal irrigated continuously with 2.25% NaOCl solution.* Results*. Periodontal temperature increase depends on the value of energy density. Means and standard deviations of temperature increases at root surfaces were below 10°C (safe threshold level) when the average energy densities delivered per second were equal to or below 4981 J/cm^2^ and 9554 J/cm^2^, respectively, for irradiations using a fiber diameter of 320 *μ*m and 200 *μ*m.* Conclusions*. Within the limitations of this study and under specific irradiation conditions, Nd:YAP laser beam may be considered harmless for periodontal tissues during endodontic applications.

## 1. Introduction

The removal of smear layer and disinfection of canals are important objectives for the success of endodontic treatments. Many methods have been proposed to achieve these objectives (irrigants, disinfecting drugs, ultrasounds, etc.). Several studies suggested the use of laser for smear layer removal and root canals disinfection: Er:YAG laser is shown to be efficient for smear layer removal from root canals [1]. Sahar-Helft et al. [2] demonstrated that smear layer removal was most effective compared to passive ultrasonic irrigation when 17% EDTA solution was activated in root canals using Er:YAG laser at low energy. Interestingly, removal of smear layer along the entire canal was similar when the laser was inserted in the coronal third or 1 mm short of the working length. This effect was not observed with ultrasonic activation or positive pressure techniques [2]. Other laser wavelengths were proposed for the removal of smear layer. Da Costa Lima et al. [3] demonstrated that Nd:YAG laser beam can also be used as an adjunct for smear layer removal with however less efficiency than the passive ultrasonic irrigation.

It is noteworthy that access of irrigants and disinfecting solutions to secondary canals and deep dentinal tubules is difficult. Schoop et al. [4] showed that Nd:YAG, diode, Er:YAG, and Er,Cr:YSGG laser beams are suitable for the disinfection of deeper layers of dentin and may constitute valuable tools in the endodontic disinfection process. Laser light can penetrate areas of canals where irrigating and disinfecting solutions cannot reach, like secondary canals and deep dentinal tubules, and also can eliminate microorganisms [5].

The Nd:YAP laser is a laser using yttrium aluminium perovskite doped with neodymium crystal as active laser medium. It is emitted in the near infrared at 1.34 *μ*m, which is close to the wavelength of the Nd:YAG laser. The Nd:YAP laser beam shows clinically interesting properties as its good absorption by dark materials and metals. The Nd:YAP is also 20 times more absorbed by water than the Nd:YAG laser [6]. Its flexible fiber optic allows delivering energy in curved root canals where the effect of the ultrasonic instrumentation is limited due to the constraining effect of the curvature of the canal. Some authors also reported that Nd:YAP laser can be successfully used for removal of the smear layer in root canals [7, 8]. However the use of this type of laser in endodontics may generate an increase in temperature and cause periodontal tissue damage. This laser has potentially many clinical applications in dentistry and specifically in endodontics; thus, the safe irradiation conditions should be clearly defined before any future clinical use.

The aim of our study is to determine the safe range of irradiation parameters of Nd:YAP laser that can be used during endodontic treatments without damaging and overheating the periodontal tissues.

## 2. Material and Methods

The study was conducted according to the Ethic Committee Recommendations of Gent University (2014/0579). Twenty-seven caries-free single-rooted adult human teeth extracted for orthodontic reasons were collected and stored before the experiments at 4°C, in a humid atmosphere, on a gauze soaked with Hepes solution (pH 7.2 at 2 mmol/liter containing 0.19 mmol/liter of natrium azide) (Hepes, Merck, Overijse, Belgium). Age of patients ranged between 45 and 60 years. External surfaces of teeth were cleaned using a scaler after which they were decoronated under water cooling at low speed (300 rpm, Isomet, Low Speed Saw, Buehler Ltd., Lake Bluff, IL) as to obtain root segments of 11 mm. Root canals were prepared and enlarged to # 45 K file according to the conventional step-back technique in order to allow for optic fibers (diameter of 200–320 *μ*m) to be placed inside all the way to the working length of 10 mm.

### 2.1. Laser Irradiation Conditions

A Nd:YAP laser (wavelength: 1340 nm, LOBEL MEDICAL SAS, Les Roches de Condrieu, France) was used. The beam emission is the pulsed mode (5, 10, and 30 Hz) predefined and imposed by the manufacturer's parameters. The pulse duration was 150 *μ*s for all predetermined irradiation parameters. The laser apparatus was only able to deliver a pulse mode with a high peak output power per pulse. The output powers were predefined by the manufacturer and vary per second of irradiation from 0.9 W (2866 J/cm^2^ for 200 *μ*m and 1120 J/cm^2^ for 320 *μ*m fiber diameter) to 10 W (31847 J/cm^2^ for 200 *μ*m and 12453 J/cm^2^ for 320 *μ*m fiber diameter). The emitted power measured by a power meter (UP19K-15S, Gentec-EO, Québec, Canada) represented 90% of the displayed power. The predefined irradiation conditions are summarized in Tables 1 and 2, respectively, for the fiber diameter of 200 *μ*m and 320 *μ*m. To facilitate the understanding and the reproducibility of our experiences, only the output powers delivered per second for each irradiation condition (predefined by the manufacturer) will be considered in our study.

### 2.2. Experimental Setup for Temperature Rise Measurements during Laser Irradiation

We followed the setup of protocols used in previous studies for the measurements of temperature increase during laser irradiation [9, 10]. The external root surfaces were covered by thermoconductor paste (Warme Leitpaste WPN 10; Austerlitz Electronic, Nuremberg, Germany) to ensure optimal contact and maximal thermal conduction between the sensor tip of the thermocouple probe and the root surface. The thermal conductivity of the paste was 0.4 cal s^−1^m^−1^K^−1^, which was comparable to the thermal conductivity of soft tissues (0.2–0.5 cal s^−1^m^−1^K^−1^) depending on hydration [11].

Each root was closely rounded by 2 probes of a K-type thermocouple (K-type thermocouples HH806AWE Omega, Manchester, UK) with a precision of 0.01°C. One of the probes was located at 1 mm from the apex and the other at 5 mm from the cervical level. Each root was immerged into a 37°C bath keeping the cervical area above the waterline as to keep water out of the canal. The increases of temperatures caused by the irradiation of the root canal walls were then recorded and analyzed. Canal irradiation was accomplished with a circular and retrograde movement from the apex until the cervical part of the canal during 10 seconds with an axial speed of 1 mm/s. Each irradiation was done in a canal irrigated continuously with 2.25% NaOCl solution. Six records were repeated for each irradiation parameter.

Temperature measurement was performed after seeing the baseline level of temperature of root surface stable during 30 s. Temperature rise was recorded every second for 180 seconds after the end of the irradiation. Temperature increases (*D*_*t*_) were calculated as the difference between the highest recorded temperatures at the root surface (*T*_*m*_) and that recorded as baseline (room temperatures = *T*_*b*_): *D*_*t*_ = *T*_*m*_ − *T*_*b*_. We did a minimum of 5 records for each irradiation condition.

The mean and the standard deviation of recorded temperatures (*D*_*t*_) for each irradiation condition were calculated. Normality tests were performed using the Kolmogorov Smirnov (KS) test.

## 3. Results

Whatever the output power used, the temperature rise after any irradiation condition needed more than 150 seconds to get back to its baseline level.

The KS normality test and Wilcoxon Signed Rank Test (*P* value, two-tailed) showed that all groups passed normality test and correspond to Gaussian Approximation (alpha = 0.05; KS distance = 0.1788; *P* value > 0.10). One-way ANOVA and post hoc tests (Newman-Keuls Multiple Comparison Test, *P* < 0.05) showed significant difference between all groups (*P* value < 0.0001; *F* 412; *R* squared 0.9924). In groups using a fiber of 320 *μ*m as diameter, the statistical difference is not significant between the means of the groups using the energy densities of 1120 J/cm^2^ and 1743 J/cm^2^. Also, for the groups using a fiber of 200 *μ*m as diameter, the statistical difference is not significant between the means of groups using the energy densities, 2866 J/cm^2^ and 4458 J/cm^2^, and between means of the energy densities of 9554 J/cm^2^ and 6369 J/cm^2^.

After 10 seconds of irradiation (irradiation speed of 1 mm/sec), means and standard deviations of temperature increases at root surfaces were below the threshold level of 10°C, considered as safe for periodontal tissue [12], when the delivered average energy densities per second were equal to or below 4981 J/cm^2^ (4 W) and 9554 J/cm^2^ (3 W), respectively, for the irradiations using a fiber diameter of 320 *μ*m and 200 *μ*m.

**Figure 1:**
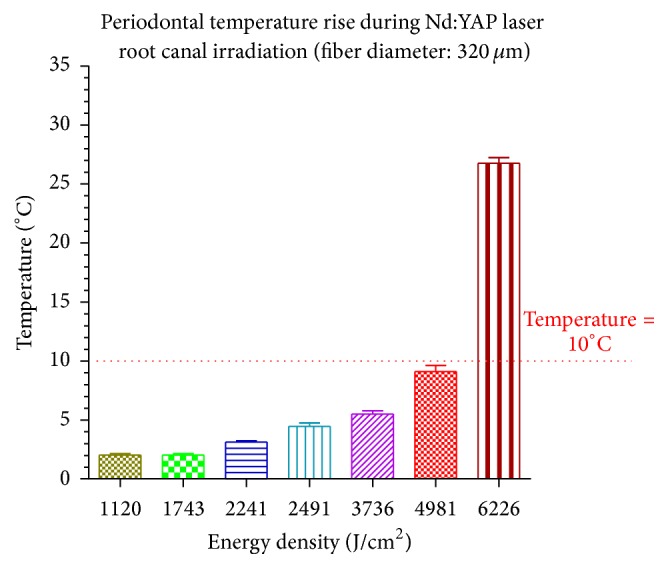
The temperature increases at root surfaces are shown in function of different average energy densities delivered per second after a total irradiation time of 10 seconds by means of fiber diameter of 320 *μ*m. When the average of delivered energy densities per second was ≤4981 J/cm^2^ (4 W), the temperature rises were below the safety level of 10°C for periodontal tissue injury.

**Figure 2:**
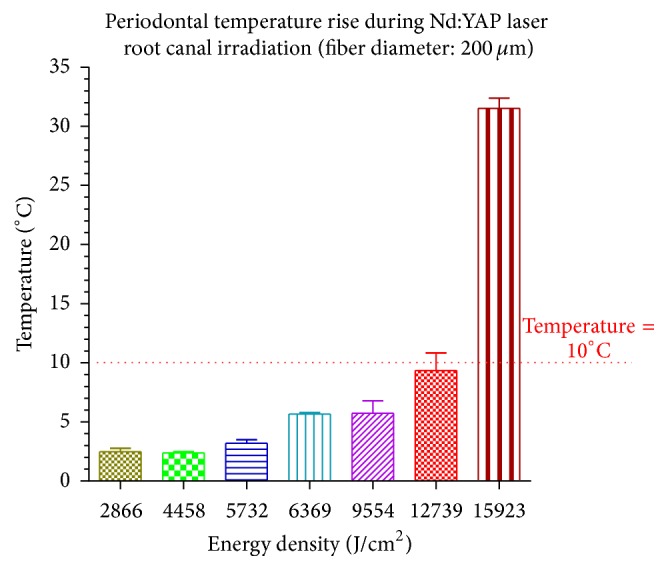
The temperature increases on root surfaces are shown in function of different average energy densities delivered per second after a total irradiation time of 10 seconds. When the average of delivered energy densities per second was ≤9554 J/cm^2^ (3 W), the temperature rises were below the safety level of 10°C for periodontal tissue injury.

Figures 1 and 2 show the temperature increases caused by 10 seconds of total irradiation time using, respectively, a fiber diameter of 200 *μ*m and 320 *μ*m.

For the use of similar irradiation parameters, each diameter of the optical fiber generated different temperature increase. The fiber with smaller diameter (200 *μ*m) generated less temperature increase than the bigger one (320 *μ*m) because of the higher distance existing between the edge of the fiber and the canal walls.

## 4. Discussion

Several studies showed some clinical applications using a Nd:YAP laser. It has been used in oral surgeries for lingual frenulectomy and frenectomy [13, 14] and for the initial treatment of periodontitis in adult [15]. In endodontic and restorative dentistry, the Nd:YAP laser was used to enhance canal cleanliness. Moshonov et al. [7] showed significant improved cleanliness into the coronal and the apical part of the root canals treated with Nd:YAP laser beam after manual preparation of the root canal with K-files and 2.5% sodium hypochlorite solution used for irrigation. Unfortunately, authors did not mention any information concerning the time consumed for canal cleaning and the detail about the way they moved the fiber into the canal (circumferential or not) and about the delivered irradiation speed given to the optical fiber (1 mm/s).

Blum and Abadie [16] pointed out that the use of the subsonic device and laser together as adjuncts showed opened tubules and the cleanest preparation was with very little debris and very small particle size. This result suggests that the laser has a potential in ensuring optimal canal cleanliness and opening tubules. The tested irradiation parameters were 260 mJ per pulse, 5 Hz, and 30 sec of irradiation each time with a constant movement of the tip when the laser is used for canal reparation or as adjunct with manual instrumentation. However, the use of Nd:YAP laser for 30 seconds without time off between successive irradiation series can induce a dangerous thermal increase, by cumulative effect, for the bone tissue according to Eriksson et al. [17].

Farge et al. [18] measured the temperature rises on root surfaces with the aim to remove the fillings of root canals by means of Nd:YAP laser. They concluded that the Nd:YAP laser used in combination with hand instrumentation can remove efficiently pulpal debris and smear layer without exceeding the temperature increase of 5.2°C. They concluded that the laser should be used into a dry canal for a total irradiation time of 1 second. The authors recommended a long resting time exceeding one minute between two irradiations in order to allow thermal relaxation.

In our study, we evaluated the harmlessness of different irradiation parameters for endodontic use in empty and unfilled root canals. We used a canal irrigant during the irradiation. Previous tests reported that using the Nd:YAP laser without irrigation can lead to faster temperature increase than with irrigant. Thus, the use of irrigant could increase slightly the irradiation working time and consequently allow longer exposure time of dentinal walls and a reduction of bone injury risk.

Any black coloration into the root canal may induce localized higher overheating. Thus, the use of the Nd:YAP laser beam for endodontic treatment into dark colored teeth should be done with precautions. In this case, it is highly recommended to reduce the total irradiation time. More studies about this subject should be done.

According to the conditions of our study we found that we can use a Nd:YAP laser beam safely with a circumferential movement with a speed of 1 mm/sec moving backward from the apex to the cervical part of the root canal (under constant irrigation flow) during 10 seconds without inducing periodontal temperature overheating exceeding the trigger temperature of 10°C if some irradiation parameters are considered. In our study, we decided to follow the root canal irradiation protocol (circumferential movement, backward movement) proposed by Gutknecht et al. [19]. Authors justified the use of circular movements' protocol to ensure that the applied laser energy is distributed as uniformly as possible on the canal walls because of the variation of the diameter of canals from the apex to the coronal part [19].

Finally further investigations should be done in order to confirm the capacity of Nd:YAP laser coupled with the irrigant to remove the smear layer of the root canal according to the safe irradiation parameters that we found.

## 5. Conclusion

Based on our study and on our in vitro irradiation conditions, the use of Nd:YAP laser for endodontic treatments may be considered as harmless for periodontal tissues under specific irradiation parameters, equal to or lower than 4981 J/cm^2^ (4 W) and 9554 J/cm^2^ (3 W), respectively, for the irradiations using a fiber diameter of 320 *μ*m and 200 *μ*m.

## Figures and Tables

**Table 1:** Different irradiation conditions are shown in function of predetermined setting of the apparatus. The average of output powers and the average of energy densities are calculated for the fiber diameter of 200 *µ*m of the Nd:YAP laser.

Setting parameter: G	Setting parameter: D	Setting parameter: C
30 Hz, 150 *μ*s per pulse	10 Hz, 150 *μ*s per pulse	5 Hz, 150 *μ*s per pulse

G+: (i) Average output power: 10 W (330 mJ per pulse) (ii) Average of energy density per second: 31847 J/cm^2^	D+: (i) Average output power: 4 W (400 mJ per pulse) (ii) Average of energy density per second: 12739 J/cm^2^	C+: (i) Average output power: 1.8 W (400 mJ per pulse) (ii) Average of energy density per second: 5732 J/cm^2^

G0: (i) Average output power: 7.5 W (250 mJ per pulse) (ii) Average of energy density per second: 23885 J/cm^2^	D0: (i) Average output power: 3 W (300 mJ per pulse) (ii) Average of energy density per second: 9554 J/cm^2^	C0: (i) Average output power: 1.4 W (280 mJ per pulse) (ii) Average of energy density per second: 4458 J/cm^2^

G−: (i) Average output power: 5 W (160 mJ per pulse) (ii) Average of energy density per second: 15923 J/cm^2^	D−: (i) Average output power: 2 W (200 mJ per pulse) (ii) Average of energy density per second: 6369 J/cm^2^	C−: (i) Average output power: 0.9 W (330 mJ per pulse) (ii) Average of energy density per second: 2866 J/cm^2^

**Table 2:** Different irradiation conditions are shown in function of predetermined setting of the apparatus. The average of output powers and the average of energy densities are calculated for the fiber diameter of 320 *µ*m of the Nd:YAP laser.

Setting parameter: G	Setting parameter: D	Setting parameter: C
30 Hz, 150 *μ*s per pulse	10 Hz, 150 *μ*s per pulse	5 Hz, 150 *μ*s per pulse

G+: (i) 330 mJ (10 W) (ii) 12453 J/cm^2^	D+: (i) 400 mJ (4 W) (ii) 4981 J/cm^2^·sec	C+: (i) 360 mJ (1.8 W) (ii) 2241 J/cm^2^

G0: (i) 250 mJ (7.5 W) (ii) 9340 J/cm^2^	D0: (i) 300 mJ (3 W) (ii) 3736 J/cm^2^	C0: (i) 280 mJ (1.4 W) (ii) 1743 J/cm^2^

G−: (i) 160 mJ (5 W) (ii) 6226 J/cm^2^	D−: (i) 200 mJ (2 W) (ii) 2491 J/cm^2^	C−: (i) 180 mJ (0.9 W) (ii) 1120 J/cm^2^
